# 泽布替尼治疗复发/难治性温抗体型自身免疫性溶血性贫血的疗效与安全性

**DOI:** 10.3760/cma.j.cn121090-20241110-00442

**Published:** 2025-04

**Authors:** 舒培 申, 辰 杨, 苗 陈, 冰 韩

**Affiliations:** 中国医学科学院，北京协和医学院，北京协和医院血液内科，北京 100730 Department of Hematology, Peking Union Medical College Hospital, Chinese Academy of Medical Sciences & Peking Union Medical College, Beijing 100730, China

## Abstract

对2022年7月至2024年1月北京协和医院10例接受泽布替尼治疗（160 mg每日2次，疗程3～6个月）并完成至少3个月随访的复发/难治性温抗体型自身免疫性溶血性贫血（wAIHA）病例进行回顾性分析。患者男2例，女8例，中位年龄63（36～76）岁，既往中位治疗时间9个月。治疗后1、3个月及随访期末（中位7个月）的总体反应率分别为30％、60％、60％，完全缓解率分别为20％、40％、40％。所有应答者中位起效时间为2（1～2）个月，未出现疾病复发或克隆演变。30％患者发生1～2级可逆性不良事件，1例有效患者于治疗8个月后死于感染性多器官功能衰竭。

## Abstract (en)

This retrospective analysis included 10 patients with relapsed/refractory warm autoimmune hemolytic anemia（wAIHA）who received zanubrutinib（treatment course: 3–6 months）and completed at least 3 months of follow-up at Peking Union Medical College Hospital from July 2022 to January 2024. The cohort included two male and eight female patients with a median age of 63 years（range: 36–76）, who had received a median of 9 months（range: 3–22）of previous therapies. At 1-month, 3-month, and final follow-up（median 7 months）, the overall response（OR）rates were 30％, 60％, and 60％, with corresponding complete response（CR）rates of 20％, 40％, and 40％, respectively. The median time to achieve a response was 2 months（range: 1–2）among the responders. No disease relapse or clonal progression was documented during follow-up. Treatment-related adverse events occurred in 30％ of the patients（all grade 1–2 reversible events）. One responder died of infectious multiple organ failure at 8 months after treatment initiation. Our results indicate that zanubrutinib provides rapid amelioration of anemia with manageable short-term safety in patients with relapsed/refractory wAIHA.

自身免疫性溶血性贫血（Autoimmune hemolytic anemia，AIHA）的特征是自身抗体和（或）补体对自体红细胞的破坏增加而引起的一组溶血性贫血，根据红细胞自身抗体对温度的反应可分为温抗体型（wAIHA）、冷抗体型（cAIHA）和混合型[1]，根据是否存在病因性疾病可分为原发性AIHA和继发性AIHA[2]。糖皮质激素单药治疗为原发AIHA的首选治疗方案，尽管可以获得70％～80％的有效率（ORR）和60％的完全缓解（CR）率，但复发率很高。二线治疗目前除了利妥昔单抗外，还包括达那唑、环孢素A、环磷酰胺、硫唑嘌呤、西罗莫司、他克莫司、脾切除等，虽有一定的疗效，但也存在耐药、复发、不良反应大等风险，且上述药物大多数缺乏大样本前瞻性研究的结果。

目前研究显示，第一代BTK抑制剂（BTKi）伊布替尼在治疗继发于慢性淋巴细胞白血病（CLL）或套细胞淋巴瘤（MCL）的AIHA方面疗效较好，不良反应较轻且可控[3]–[4]。还有一些小样本报道伊布替尼治疗原发AIHA取得了一定的疗效。作为不同于伊布替尼的第二代BTKi，泽布替尼在治疗CLL和MCL中显示了优于伊布替尼的疗效。然而，目前关于泽布替尼在原发性AIHA方面还未见国内外文献报道。本研究回顾性分析了10例复发/难治性wAIHA患者应用泽布替尼治疗的临床资料，讨论疗效及安全性。

## 对象与方法

1. 研究对象：回顾性收集了2023年2月至2024年1月在北京协和医院诊断为复发/难治性wAIHA且应用泽布替尼治疗的病例资料。纳入标准：①18岁以上；②在全剂量糖皮质激素治疗（1 mg/kg类固醇激素治疗至少1个月）后无反应或复发，且糖皮质激素在泽布替尼治疗前稳定剂量至少1个月，患者可以接受过其他二线治疗，如果接受，需在开始泽布替尼治疗前至少1个月停药；③泽布替尼治疗前的基线肝肾功能（丙氨酸转氨酶、天冬氨酸转氨酶、肌酐）低于正常范围上限的2倍；④已使用泽布替尼单药治疗至少3个月，泽布替尼治疗后随访至少3个月。本研究经过了北京协和医院伦理委员会的批准（批件号：K7906），纳入患者均签署知情同意书。

通过查阅住院病历、门诊记录及患者提供的当地医院化验结果进行数据收集。收集患者泽布替尼治疗前基线的社会人口学资料，病史，既往输血情况，血常规（含网织红细胞），生化（肝、肾、甲状腺功能，铁蛋白、转铁蛋白、转铁蛋白饱和度，叶酸，维生素B_12_），HAV、HBV、HCV、EB病毒（EBV）、巨细胞病毒（CMV）、HIV和细小病毒B19，凝血功能，直接抗人球蛋白试验（DAT），超声心动，腹部超声，骨髓穿刺、活检，染色体核型，骨髓增生异常综合征（MDS）、骨髓增殖性肿瘤（MPN）相关基因检测等。同时采集泽布替尼治疗后1、3个月及随访期末的症状、体征、输血情况、血常规（含网织红细胞）、生化（肝、肾功能，铁蛋白，叶酸，维生素B_12_）及6个月的骨髓穿刺、活检、染色体结果，统计治疗相关并发症及转归情况。

2. 治疗方案：泽布替尼160 mg每日2次，无论是否起效，至少服用3个月。对于有反应的患者，泽布替尼持续服用6个月后停用。若之前正在使用激素治疗，可继续使用，但剂量应低于泼尼松20 mg/d（其他激素按此剂量折算），且无其他合并用药。

3. 诊断和疗效判断：wAIHA的诊断基于第1次国际共识会议建议的诊断标准[3]，在诊断前需与其他可能的疾病进行鉴别诊断。所有患者接受治疗前均查血常规、生化、免疫指标、肿瘤标志物、CT、B超、心电图、骨髓涂片、活检、染色体核型和基因突变等，对潜在疾病（如胸腺瘤、结缔组织病、病毒感染、血液肿瘤或实体瘤）进行进一步筛查，且排除MDS或急性髓系白血病等其他血液疾病。

疗效标准参照AIHA第1次国际共识会议建议[3]。CR：HGB>110 g/L；部分缓解（PR）：HGB接近正常，或如果基线HGB<60 g/L，则至少增加了20 g/L且治疗最后7 d没有输血；无效（NR）：不符合CR或PR的疗效标准。复发定义为治疗反应由CR转为PR或CR/PR转为NR。总体反应（OR）为CR+PR。

安全性分析包括评估不良事件的发生率和严重程度，报告治疗期间发生或恶化的所有不良事件以及后来发生但被认为与泽布替尼使用有关的不良事件。不良事件参考美国国立癌症研究所的不良事件常用术语标准5.0版（Common terminology criteria for adverse events, CTCAE v5.0）进行评估与描述。

4. 统计学处理：使用SPSS26.0软件进行统计分析。计量资料以中位数（范围）、计数资料以例数（构成比）进行描述。符合正态分布的计量资料以均数±标准差表示，两组数据比较采用配对*t*检验；非正态分布计量资料以中位数和范围表示，两组数据比较采用非参数秩和检验。*P*<0.05则认为两组数据间差异具有统计学意义。

## 结果

1. 患者特征：最终纳入10例接受泽布替尼治疗的wAIHA患者，其中男2例，女8例，中位年龄63（36～76）岁。8例诊断难治性wAIHA，2例诊断为复发wAIHA。泽布替尼治疗前中位HGB水平为76（59～95）g/L，中位WBC为7.5（1.8～19.0）×10^9^/L，中位PLT为233（105～369）×10^9^/L，中位网织红细胞绝对值为371.6（107.5～732.3）×10^9^/L，中位网织红细胞百分比为7.6％（4.2％～46.4％），中位总胆红素为106.2（8.7～129.9）µmol/L，中位间接胆红素为50.8（4.9～118.2）µmol/L，中位血清铁蛋白水平为305（121～3057）µg/L。所有患者Coombs试验均阳性。

既往接受治疗的中位线数为4（2～8）线，既往治疗的中位时间为9（3～22）个月。既往治疗方案包括：2例患者使用糖皮质激素治疗；2例患者使用糖皮质激素+免疫抑制治疗+利妥昔单抗+静脉注射丙种球蛋白（IVIG）治疗；1例患者使用糖皮质激素+免疫抑制治疗+利妥昔单抗+IVIG+脾切除治疗；1例患者使用糖皮质激素+免疫抑制治疗+利妥昔单抗治疗；1例患者使用糖皮质激素+免疫抑制治疗+IVIG治疗；3例患者使用糖皮质激素+免疫抑制治疗。

2. 疗效：泽布替尼中位使用时间为6（3～6）个月，中位随访时间为7（6～9）个月。在随访1个月时，总体反应率（ORR）为30％，CR率为20％；在随访3个月时，ORR为60％，CR率为40％，在随访期末时，ORR为60％，CR率为40％，所有患者达到OR和CR的中位时间均为2（1～2）个月。

患者治疗前中位HGB为76（59～95）g/L，治疗1个月时的HGB为99（63～131）g/L，较治疗前无明显改善（*P*＝0.478）；3个月时的HGB为112.5（63～133）g/L，较治疗前明显改善（*P*＝0.015）。随访期末HGB为110.5（63～140）g/L，较治疗前明显改善（*P*＝0.020）。治疗1个月、3个月、随访期末时，WBC、PLT、铁蛋白水平、网织红细胞绝对值、总胆红素、直接胆红素同治疗前相比差异无统计学意义（均*P*>0.05）。随访期末时，达到OR的患者（6例），中位HGB升高30（23～62）g/L，而中位WBC、PLT未见明显变化（表1）。

**表1:** 10例复发/难治性温抗体型自身免疫性溶血性贫血患者泽布替尼治疗前后的血常规/生化指标变化

指标	基线	治疗1个月	*P*值	治疗3个月	*P*值	随访期末	*P*值
HGB［g/L，*M*（范围）］	76（59～95）	99（63～131）	0.478	112.5（63～133）	0.015	110.5（63～140）	0.020
Ret［×10^9^/L，*M*（范围）］	371.6（67.5～732.3）	369.0（72.2～651.6）	0.851	365.3（70.6～677.8）	0.886	363.8（81.1～701.9）	0.903
WBC［×10^9^/L，*M*（范围）］	7.50（3.60～19.00）	8.70（1.77～23.27）	0.762	7.37（3.59～18.27）	0.871	7.42（3.19～18.15）	0.919
PLT［×10^9^/L，*M*（范围）］	233（105～969）	193（103～532）	0.402	177（120～580）	0.275	178（97～515）	0.301
ALT［U/L，*M*（范围）］	60（13～101）	53（15～99）	0.563	49（15～97）	0.439	40（15～102）	0.252
TBIL［µmol/L，*M*（范围）］	106.2（8.2～129.9）	100.3（11.3～132.1）	0.348	91.3（7.7～127.6）	0.334	78.5（8.0～113.4）	0.182
DBIL［µmol/L，*M*（范围）］	17.6（3.3～40.5）	17.7（2.9～37.6）	0.941	15.3（3.3～42.1）	0.836	18.0（2.1～45.5）	0.793
SF［µg/L，*M*（范围）］	305（11～3 057）	336（23～1 815）	0.362	352（18～2 323）	0.241	297（14～2 317）	0.334
Cr［µmol/L，*M*（范围）］	63（53～101）	75（61～114）	0.342	57（44～92）	0.617	60（51～103）	0.769

**注** Ret：网织红细胞绝对值；TBIL：总胆红素；DBIL：直接胆红素；SF：血清铁蛋白；Cr：血肌酐。*P*值为与基线比较

3. 不良反应：随访期内，3例发生不良反应。1例患者因血小板减少、皮下出血和紫癜停药2周，停药后不良反应逐渐消失，后恢复了泽布替尼的使用。1例患者在治疗2个月时出现了丙氨酸转氨酶的升高及高尿酸血症，在对症治疗后症状缓解。1例患者在使用泽布替尼1个月时出现了恶心，未经特殊治疗好转。与药物相关的不良反应均为1～2级，未见其他肝肾功能损害、过敏及其他不良反应。无导致停药或死亡的不良反应。

4. 复发与最终结局：至随访期末时，所有获得过OR的患者中，没有患者复发。1例NR患者（共使用泽布替尼3个月）在随访8个月时，因隐球菌性脑膜炎引起感染性多器官功能衰竭死亡，判断与泽布替尼的使用无关。

## 讨论

激素常规治疗AIHA疗效较高，但复发率高，长期使用激素还可能带来许多激素相关的不良反应。而目前各种二线治疗方案都存在局限性，存在未被满足的临床需求。

BTK在B细胞受体（BCR）信号通路中起到关键的作用。BTK可影响信使分子的产生，而信使分子可异常激活BCR信号通路，并将B细胞转化为介导自身免疫性疾病的自身反应性B细胞[5]–[6]。根据作用机制的不同，BTKi可以分为共价抑制剂和非共价抑制剂。共价抑制剂通过共价键结合野生型或突变型Cys481残基，非共价抑制剂通过氢键或疏水相互作用等非共价力占据ATP结合口袋或BTK的特定H3口袋，从而有效和持续抑制BTK的酶活性[7]–[8]。目前有三种不可逆的共价BTKi（伊布替尼、阿卡替尼、泽布替尼）已经被批准用于治疗淋巴系统恶性肿瘤，而替拉鲁替尼、奥布替尼等其他不可逆的共价BTKi正在进行相关临床研究[9]。

在AIHA治疗领域，伊布替尼的临床应用已有一定报道。Manda等[10]报道了1例无症状CLL继发AIHA患者，经伊布替尼治疗后，HGB水平显著上升，临床溶血症状完全消失。Rogers等[11]研究发现，伊布替尼治疗的复发/难治性CLL/小淋巴细胞淋巴瘤患者中，紧急AIHA的发生率较未使用伊布替尼的患者更低。一项Ⅲ期临床试验也发现在治疗B细胞CLL期间，伊布替尼组没有患者发生AIHA，而仅接受苯丁酸氮芥治疗的患者中有2％发生了AIHA[12]。方力维等[13]研究表明，伊布替尼对原发性复发/难治性AIHA患者有疗效，且不良反应较轻。这些有限的关于伊布替尼的研究数据，表明BTKi可能对原发性复发/难治性AIHA患者均有一定的疗效，且不良反应可控。Yan等[14]在2023 EHA上报道了第二代BTKi奥布替尼在免疫性血栓性血小板减少症（ITP）中的疗效，33例患者按1∶1的比例随机接受口服奥布替尼30 mg或50 mg，每天1次，共治疗24周，总体有效率为36.4％，持久缓解率为27.3％，且安全性和耐受性良好。AIHA与ITP均为自身免疫机制导致的血细胞减少[15]，其发病机制有一定的相似之处，该研究的结果也为BTKi在这类疾病中的应用提供了佐证。

泽布替尼是第二代不可逆BTKi，已经被证实在治疗B细胞淋巴恶性肿瘤方面有较好的疗效。但目前关于泽布替尼在复发/难治性AIHA的疗效及安全性尚未见报道。本研究回顾性分析了泽布替尼治疗复发/难治性AIHA的疗效及安全性，结果显示，泽布替尼治疗1个月时，ORR为30％，CR率为20％；3个月时的ORR为60％，CR率为40％；在随访期末时，ORR为60％，CR率为40％，所有患者达到OR和CR的中位时间为2（1～2）个月。患者治疗3个月和随访期末时的HGB均较治疗前明显改善。在随访期末，获得OR的患者，中位HGB较基线升高30（23～62）g/L。这些结果与其他研究者在其他类型BTKi中的结果类似[10],[13]。说明泽布替尼通过阻断产生抗体的通路，减少了自身抗体对红细胞的攻击，从而改善了造血。关于BTKi在ITP中作用机制研究较少。Yan等[14]开展的小鼠模型研究表明，奥布替尼通过促进B细胞凋亡和抑制单核细胞来源巨噬细胞的吞噬活性来抑制自身免疫机制对血细胞的攻击，从而提高血小板计数，这些为BTKi的作用机制提供了理论基础。

至随访期末时，没有患者复发。目前仅有方力维等[13]的研究探究了伊布替尼治疗复发/难治性原发性AIHA的复发情况，但其样本量仅为2例，2例患者均未复发。仍需样本量更大的队列研究来对BTKi治疗AIHA的复发率和最终转归进行探究。

泽布替尼的常见不良反应包括中性粒细胞减少、血小板减少、贫血、出血、心律失常、上呼吸道感染和肌肉骨骼疼痛等。本研究中，仅1例患者出现血细胞减少症、皮下出血和紫癜，未观察到白细胞减少及其他不良反应，可能与样本量小、随访时间短有关。1例患者死于隐球菌性脑膜炎，因其死亡时已停用泽布替尼6个月，判断与泽布替尼使用无关。

本研究为回顾性研究，存在选择偏倚、数据缺失等问题，且样本量少、随访时间短，但初步结果显示泽布替尼对复发难治性AIHA具有一定的疗效，CR率高，不良反应轻微，为后续开展前瞻性临床研究提供了依据。目前，我们已注册相关临床试验（NCT05922839），并招募了8例患者。国际上，类似研究也在积极开展，如一项单臂Ⅱ期临床研究（NCT03827603）将复发/难治性AIHA患者分两个阶段进行试验，第一阶段给予伊布替尼联合利妥昔单抗治疗6个月，第二阶段维持伊布替尼单药治疗，结果将与利妥昔单抗单药治疗或联合化疗治疗的患者进行历史对照。此外，伊布替尼治疗复发/难治性AIHA的Ⅱ期临床研究（NCT04398459）以及评估阿卡替尼治疗由CLL继发的复发/难治性AIHA患者的疗效和安全性的Ⅱ期临床研究也正在筹备阶段（NCT04657094）。
